# Serum biomarker panel diagnostics in pancreatic ductal adenocarcinoma: the clinical utility of soluble interleukins, IFN-γ, TNF-α and PD-1/PD-L1 in comparison to established serum tumor markers

**DOI:** 10.1007/s00432-022-04112-z

**Published:** 2022-06-23

**Authors:** Klara Dorman, Miriam Gerckens, Stephan Kruger, Kimberly Krueger, Zsuzsanna Mayer, Alexander Rupp, Danmei Zhang, Lena Weiss, C. Benedikt Westphalen, Michael Haas, Michael Guenther, Steffen Ormanns, Frank Klawonn, Jens Werner, Michael von Bergwelt-Baildon, Volker Heinemann, Stefan Boeck, Stefan Holdenrieder

**Affiliations:** 1 Department of Internal Medicine III and Comprehensive Cancer Center, Klinikum Grosshadern, Ludwig-Maximilians-University of Munich, Marchioninistr. 15, 81377 Munich, Germany; 2 German Cancer Consortium (DKTK), Partner Site Munich, Munich, Germany; 3 Munich Biomarker Research Center, Institute of Laboratory Medicine, German Heart Center, Technical University of Munich, Munich, Germany; 4 Institute of Pathology, Faculty of Medicine, Ludwig-Maximilians-University of Munich, Munich, Germany; 5 Department of Computer Science, Ostfalia University, Wolfenbüttel, Germany; 6 Biostatistics Research Group, Helmholtz Centre for Infection Research, Braunschweig, Germany; 7 Department of General, Visceral and Transplant Surgery, Ludwig-Maximilians-University of Munich, Munich, Germany; 8 Center for the Evaluation of Biomarkers, CEBIO, Munich, Germany

**Keywords:** Biomarker, Cytokine, Interleukin, Pancreatic cancer, PD-1/PD-L1

## Abstract

**Purpose:**

Novel biomarkers to better predict outcome and select the best therapeutic strategy for the individual patient are necessary for pancreatic ductal adenocarcinoma (PDAC).

**Methods:**

Using a panel assay, multiple biomarkers (IFN-γ, IL-10, IL-6, IL-8, TNF-α, CEA, CA 19–9, CYFRA 21–1, HE4, PD-1 and PD-L1 levels) were measured in serum samples of 162 patients with resected, locally advanced and metastatic PDAC in this retrospective single-center study. Optimal cut-off values to differentiate prognostic subgroups with significantly different overall survival (OS) were determined by receiver operator characteristics and Youden Index analysis. Marker levels were assessed before the start of chemotherapy and correlated with OS by univariate and multivariate Cox analysis.

**Results:**

Median OS for resected patients was 28.2 months, for locally advanced patients 17.9 months and for patients with metastatic disease 8.6 months. CYFRA 21–1 and IL-8 discriminated metastatic from locally advanced patients best (AUC 0.85 and AUC 0.81, respectively). In univariate analyses, multiple markers showed prognostic relevance in the various subgroups. However, multivariate Cox models comprised only CYFRA 21–1 in the resected group (HR 1.37, *p* = 0.015), IL-10 in locally advanced PDAC (HR 10.01, *p* = 0.014), as well as CYFRA 21–1 and CA 19–9 in metastatic PDAC (*p* = 0.008 and *p* = 0.010) as an independent prognostic marker for overall survival.

**Conclusion:**

IL-10 levels may have independent prognostic value in locally advanced PDAC, whereas CYFRA 21–1 levels are prognostic after PDAC surgery. CYFRA 21–1 and IL-8 have been identified to best discriminate metastatic from locally advanced patients.

**Supplementary Information:**

The online version contains supplementary material available at 10.1007/s00432-022-04112-z.

## Introduction

Pancreatic ductal adenocarcinoma (PDAC) remains one of the most common and deadliest cancers. A 5-year overall survival rate of 10% (Siegel et al. 2021) and increasing incidence rates (Rahib et al. 2014) emphasize the unmet clinical need for more impactful therapeutic and diagnostic options. While combination chemotherapies have prolonged progression-free survival and overall survival (OS), they are often accompanied by significant side effects (Conroy et al. 2011; Von Hoff et al. 2013). Biomarkers to better stratify which patients benefit from a certain therapy, to allow early response assessment, or to identify patients with better or worse prognosis, are subject to ongoing research. So far, the biomarker best established as part of routine PDAC care is carbohydrate antigen 19–9 (CA 19–9) (Boeck et al. 2006). It has been shown that baseline CA 19–9 levels as well as CA 19–9 kinetics under treatment are significant prognostic factors in patients with advanced PDAC (Boeck et al. 2010; Haas et al. 2013; Chiorean et al. 2016). In patients with resectable PDAC, a preoperative constellation of high CA 19–9 combined with CEA (carcinoembryonic antigen) and CA 125 (carbohydrate antigen 125) elevation is associated with poor surgical outcome (Liu et al. 2015). The diagnostic value of CEA alone as a predictor of advanced PDAC could be demonstrated as well and diagnostical cut-off values have been determined (van Manen et al. 2020). However, optimal cut-off values for common biomarkers to predict the prognosis within a pancreatic cancer stage are yet to be defined. Although used to a lesser extent in clinical routine, CYFRA 21–1 (cytokeratin 19 fragment 21–1) has been identified as an independent predictor for OS in advanced PDAC while also being a significant marker for objective treatment response during the course of treatment (Boeck et al. 2013). Besides diagnostic and prognostic value, another important aspect of the establishment of biomarkers is accessibility, making blood-based biomarkers more attractive than tissue-based markers, as it allows for convenient evaluation of multiple time points without relevant additional risk to the patient. The development of various panel diagnostics has opened new possibilities to assess multiple biomarkers in one run, requiring only a small amount of sample material (Song et al. 2019).

In this retrospective, single-center biomarker study, multiple investigational biomarkers were measured in serum samples from 162 patients with resected, locally advanced or metastatic PDAC. The aims of the study were to identify novel diagnostic and prognostic biomarkers as well as cut-off values thereof, to correlate the biomarker serum levels with each other, and to investigate differences in biomarker serum levels and their discriminatory power between resected, locally advanced, and metastatic PDAC patients. Besides the already well-established biomarkers CA 19–9, CEA and CYFRA-21, we chose to investigate protein markers such as HE4, soluble PD-1 and PD-L1. Furthermore, we evaluated a panel of proinflammatory cytokines that have not been extensively examined in PDAC patients receiving chemotherapy yet but are thought to play an important role in the biology of PDAC, a disease associated with a pronounced inflammatory tumor microenvironment (Padoan et al. 2019).

## Patients and methods

### Patient population and treatment

Patients with histologically confirmed resected, locally advanced and metastatic PDAC treated at the LMU outpatient clinic between 2011 and 2020 were considered eligible for this study (figure S1). Patients with secondary neoplasia or who had received previous systemic treatment for PDAC were excluded. Commencement of neoadjuvant, adjuvant or palliative chemotherapy within 30 days after study inclusion, as well as the availability of pre-therapeutic serum samples were mandatory. The study has been approved by the local ethics committee of the Ludwig-Maximilians-University Munich (approval number 284–10); all patients had given written informed consent before any study-specific procedure was performed.

### Sample collection and assays

Venous blood samples were collected in gel-separation tubes (Sarstedt, Nümbrecht, Germany) before initiation of chemotherapy (either in adjuvant, neoadjuvant or palliative intent). The samples were centrifuged at 3000 rounds per minute (rpm) for 10 min at room temperature; afterwards the serum was separated, aliquoted and frozen at – 80 °C. Samples were only thawed prior to analysis. CA 19–9, CEA, CYFRA 21–1 and HE4 were measured by the electrochemiluminescence immunoassay Elecsys^®^ and the Cobas e 411 Analzyer (Roche Diagnostics, Penzberg, Germany) as described previously (Boeck et al. 2013). For measurement of the serum cytokines IFN-γ, IL-1β, IL-2, IL-4, IL-6, IL-8, IL-10, IL-12p70, IL-13, and TNF-α the commercially available Multi-Spot Assay V-PLEX Plus Proinflammatory Panel 1 Human Kit and the Mesoscale Quickplex SQ 120 (both obtained from Meso Scale Diagnostics, LLC, Rockville, USA) were used. The measurements were performed according to the manufacturer’s instructions.

Soluble PD-1 and PD-L1 were measured by Sandwich-ELISA using the Human PD-1 and Human PD-L1 DuoSet ELISA Development Kits (R&D Systems, Minneapolis, USA) and the Mesoscale Quickplex SQ 120 platform In brief, quickplex 96-well standard plates were incubated with 25 µl/well capture antibodies (concentration 2 µg/ml for PD-1 and 4 µg/ml for PD-L1), sealed and incubated overnight. On the following day, the plates were washed three times (200 µl/well 0.05% Tween^®^20). Next, 150 µl PBS with 5% BSA were added as a blocking agent, the plate was then sealed and shaken at 500 rpm for 1 h. After another washing step, 25 µl of calibrators or patient samples were added and incubated for 2 h while being shaken at 500 rpm. The calibration curve consisted of 1:4 dilutions of the standard ranging from 30 ng/ml to 7 pg/ml. For the PD-1 assay, diluent 2 was used for dilution, and for the PD-L1 assay PBS with 1% BSA was used. The samples were added undiluted. After a further washing step as described above, 25 µl/well of unlabeled detection antibodies were added (concentration 400 ng/ml for PD-1 and 100 ng/ml for PD-L1). The plates were sealed and incubated for 2 h under shaking conditions. Afterwards, the plates were washed once more, and 25 µl streptavidin-sulfo-tag antibodies were added and incubated for 2 h. After another washing step, 150 µl/well MSD Gold Read Buffer A was added and chemiluminescent measurement was conducted by the Mesoscale Quickplex SQ 120 reader. Acquired data were analysed with the software Discovery Workbench 4.0.12.

### Study design and statistical analysis

The primary goal of this retrospective biomarker study was to evaluate the prognostic value of 16 serological biomarkers with regard to OS in patients with resected, locally advanced and metastatic PDAC. OS was defined as the time interval between study inclusion until death from any cause. Median follow-up was 52.1 months; the observations were censored for patients alive at a pre-defined time point (January 31st, 2020). Secondary study endpoints included the quantification limits and distribution of biomarkers, correlation of the biomarker serum levels with each other, evaluation of differences and discriminatory power in biomarker serum levels between the three study groups regarding the stage of disease and definition of relevant cut-offs.

The correlation between biomarker serum levels and patient age was estimated by Spearman’s rank-order correlation. Correlation of the biomarker serum levels with each other were also calculated with Spearman’s rank-order correlation and visualized in a heatmap. The differences in biomarker serum levels between the three study groups were tested by Kruskal–Wallis test and in regard to the ability to differentiate between the study groups by the area under the receiver operator characteristics (ROC) curve. A higher area under the curve (AUC) marks a higher discriminatory power. A 95% confidence interval (CI) not including the value 0.5 was considered a significant result.

By univariate Cox regression analysis, interval scaled biomarker serum levels were examined for prognostic relevance. Optimal biomarker serum level cut-off values that differentiate between a better or poorer outcome than group-specific median OS were calculated by ROC and Youden Index. Based on these cut-off values the study groups were divided into two groups and OS was estimated by the Kaplan–Meier method; differences in the survival curves were calculated by log-rank test (with a p-value of < 0.05 regarded as statistically significant). Parameters with statistical significance in the univariate analysis were included in multivariate Cox regression models.

## Results

### Patient characteristics

Ninety-five male and 67 female patients with pancreatic cancer from our high-volume comprehensive cancer center were included in this study. Baseline characteristics are summarized in Table 1: the majority of included patients (*n* = 103, 64%) presented with metastatic disease, while 20 patients (12%) had locally advanced PDAC, and 39 patients (24%) had undergone curative-intent surgical tumor resection before inclusion in this biomarker study (figure S2). The median time interval between tumor resection and measurement of serum markers was 54 days; all patients with resected PDAC received adjuvant chemotherapy, in most cases (92%) single-agent gemcitabine. Out of 20 patients with locally advanced PDAC, 9 were treated with palliative chemotherapy, and 11 received neoadjuvant chemotherapy, followed by secondary tumor resection in 8 patients. Systemic treatment for locally advanced PDAC most frequently consisted of 5-FU-based regimens (75%). The majority of patients with stage IV PDAC had synchronous metastatic disease (87%) and most received palliative chemotherapy (97%). 5-FU-based regimens were most common (44%), followed by gemcitabine-based regimens (32%) and gemcitabine monotherapy (24%). Only three patients with metastatic PDAC received ‘neoadjuvant’ chemotherapy and palliative resection of the primary tumor as an individualized therapeutic concept. Median follow-up after inclusion in this study was 52.1 months. Patients with resected PDAC had a median OS of 28.2 months, while median OS in locally advanced and metastatic PDAC patients were estimated at 17.9 and 8.6 months, respectively.

**Table 1:** Patient characteristics at time of serum sampling (*n* = 162)

Stage of disease (at time of serum sampling)	Resected	Locally advanced	Metastatic
Patients			
Number of patients	39	20	103
Age (in years)			
Median	66.0	66.5	63.0
Range	41–77	46–83	29–86
Gender			
Male	24 (62%)	7 (35%)	64 (62%)
Female	15 (38%)	13 (65%)	39 (38%)
Performance Status			
ECOG 0	13 (33%)	12 (60%)	29 (28%)
ECOG 1	12 (31%)	5 (25%)	34 (33%)
ECOG 2	0 ( 0%)	0 ( 0%)	7 ( 7%)
ECOG 3	0 ( 0%)	0 ( 0%)	1 ( 1%)
Missing	14 ( 36%)	3 (15%)	32 (31%)
Tumor localization			
Caput	32 (82%)	16 (80%)	46 (45%)
Corpus, Cauda	7 (18%)	4 (20%)	54 (52%)
Missing	0 ( 0%)	0 ( 0%)	3 ( 3%)
Tumor histology			
Ductal origin	37 (95%)	19 (95%)	97 (94%)
Acinar cell	1 ( 3%)	0 ( 0%)	2 ( 2%)
Missing	1 ( 3%)	1 ( 5%)	4 ( 4%)
Time of metastasis			
Synchronous			90 (87%)
Metachronous			13 (13%)
UICC TNM classification			
UICC IIA	12 (31%)		
UICC IIB	26 (67%)		
UICC III	1 ( 3%)		
Resection status			
R0	28 (72%)		
R1	10 (26%)		
R2	1 ( 3%)		
Chemotherapy after serum sampling			
Chemoradiotherapy	1 ( 3%)	0 ( 0%)	0 ( 0%)
Gemcitabine monotherapy	36 (92%)	3 (15%)	25 (24%)
Gemcitabine-based combination therapy	1 ( 3%)	2 (10%)	33 (32%)
5-FU-based combination therapy	1 ( 3%)	15 (75%)	45 (44%)
Deceased			
Number of patients	25 (64%)	17 (85%)	99 (96%)

### Quantification limits and distribution of biomarkers

Of the serological biomarkers investigated in this study, the interleukins IL-1β, IL-2, IL-4, IL-12p70 and IL-13 were not included in the further analysis due to more than 95% of measured values lying below the lower level of quantification of the assay used (for details see table S1). The boxplot distribution of the biomarker serum levels of IFN-γ, IL-10, IL-6, IL-8, TNF-α, CEA, CA 19–9, CYFRA 21–1, HE4, PD-1 and PD-L1 is illustrated within figure S3. The right skewness is reduced by logarithmic transformation, however, there is still considerable deviance from a normal distribution.

### Correlation of serological biomarkers and group differences

**Fig. 1:**
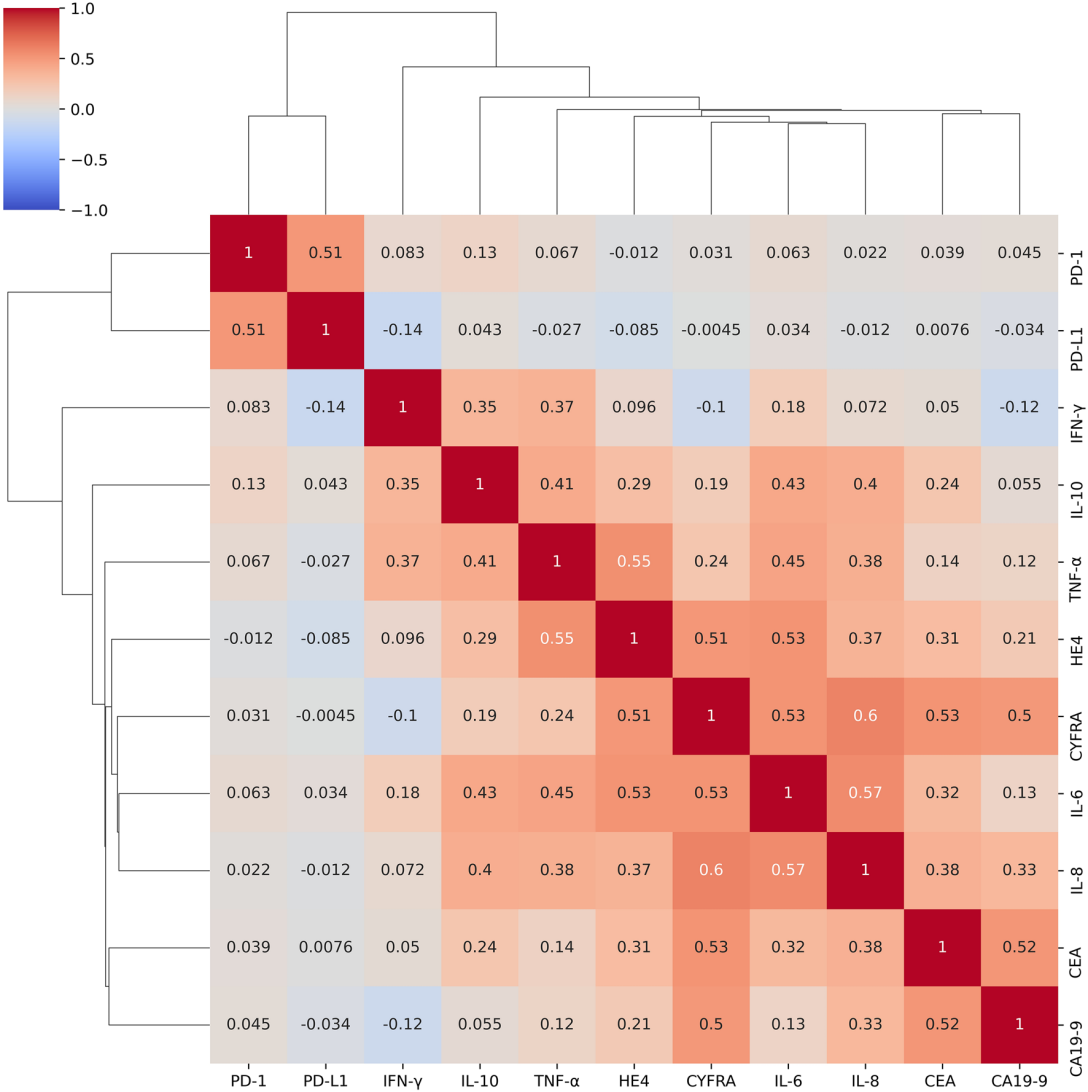
Spearman’s correlation coefficient (R) between serum biomarker levels in a color-coded heatmap with hierarchical clustering

**Fig. 2:**
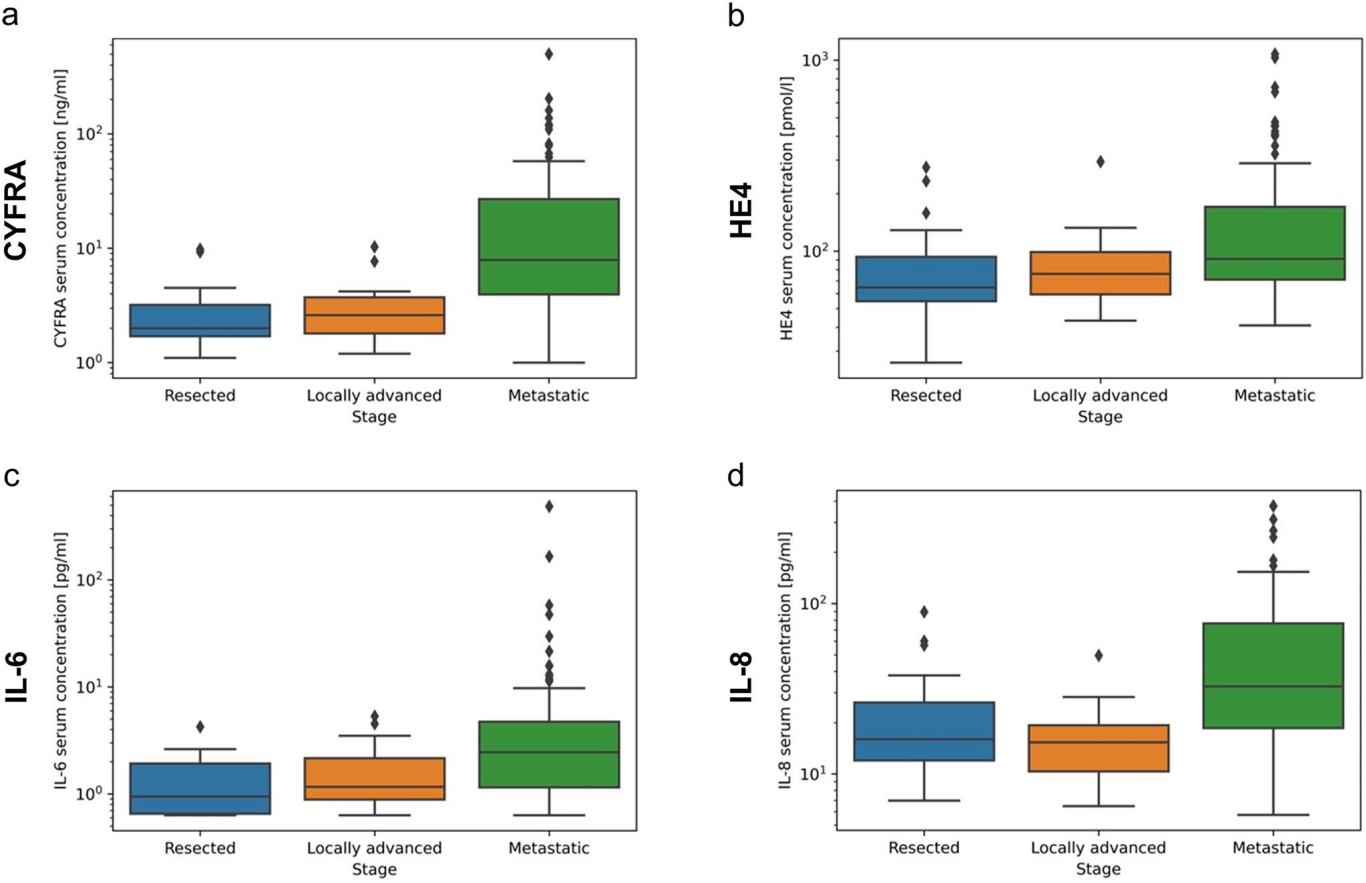
Distribution of serum concentrations of CYFRA (**a**), HE4 (**b**), IL-6 (**c**) and IL-8 (**d**) by patient subgroups in boxplot diagrams using logarithmic y-axis scales

Among the biomarkers that were evaluated further, a Spearman's correlation analysis revealed a correlation between CYFRA 21–1 and IL-8, IL-6 and IL-8, as well as TNF-α and HE4 (*R* = 0.60, 0.57, and 0.55, respectively, *p* < 0.001 for all, Fig. 1). Furthermore, a significant correlation between age and TNF-α, as well as age and HE4 was observed (*R* = 0.17, *p* = 0.03 and *R* = 0.25, *p* = 0.001, respectively, figure S4). As expected, Kruskal–Wallis test and Dunn's test showed a significant difference in median serum levels of the more established PDAC biomarkers CA 19–9 and CEA between the group of metastatic disease and the post-resection levels after PDAC surgery (data not shown, *p* < 0.001 for both biomarkers). While there was no substantial difference in median CYFRA 21–1 serum levels between the locally advanced group and the resected group, a significant difference was observed between the pre-therapeutic CYFRA 21–1 serum levels of patients with metastatic and locally advanced PDAC, as well as the metastatic and resected group (*p* < 0.001 for both comparisons, Fig. 2a). Further, IL-8 serum levels differed between metastatic PDAC patients compared to locally advanced and resected patients (*p* < 0.001 for both comparisons, Fig. 2d), while IL-6 and HE4 serum levels only differed significantly when comparing the metastatic group with the resected group (*p* < 0.001 for both comparisons, Fig. 2b, c). The differences in median PD-1 and PD-L1 serum levels were not different across all three groups (data not shown).

**Fig. 3:**
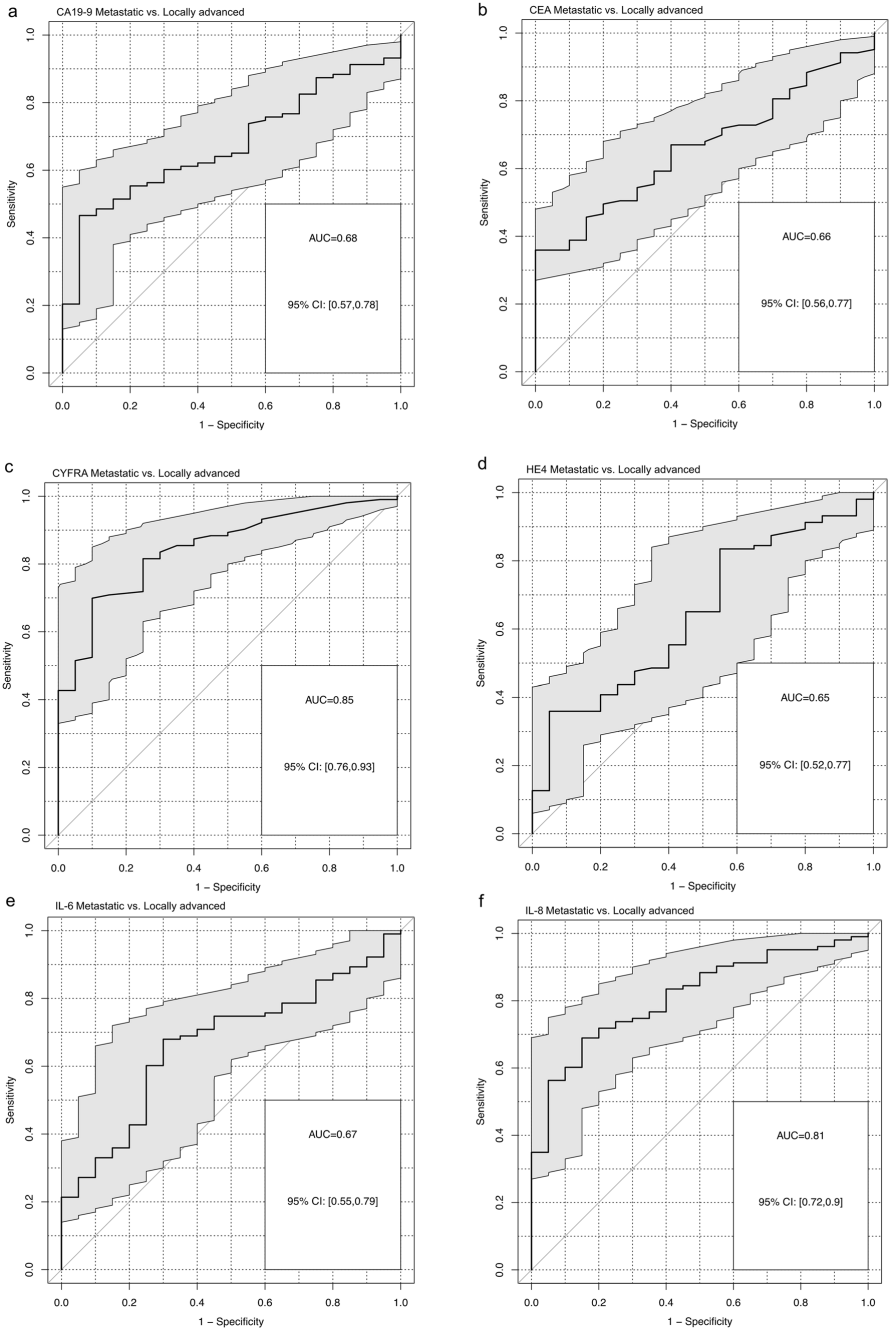
Receiver-Operator-Characteristics (ROC) Curves und Area-under-the-Curves (AUC) analysis to illustrate potential differences in serum concentrations of CA19-9 (**a**), CEA (**b**), CYFRA 21–1 (**c**), HE4 (**d**), IL-6 (**e**) and IL-8 (**f**) between patients with metastatic pancreatic cancer and locally advanced pancreatic cancer

The biomarkers were also analyzed for their ability to differentiate between the patient groups by ROC analysis. Based on the AUC, CYFRA 21–1, CEA, CA 19–9, HE4, IL-6, and IL-8 discriminated between metastatic and resected patients, as well as metastatic and locally advanced patients (Fig. 3). Especially CYFRA 21–1 and IL-8 both discriminated the metastatic group from the locally advanced group well (AUC 0.85, 95% CI 0.76–0.93 and AUC 0.81, 95% CI 0.72–0.9, respectively, Fig. 3).

### Univariate analysis for a correlation of serological biomarkers with prognosis

In the group of resected PDAC patients, univariate Cox regression revealed a significant correlation between higher IL-10 and CYFRA 21–1 serum levels and poorer OS (HR 1.43, 95% CI 1.09–1.87, *p* = 0.010 and HR 1.49, 95% CI 1.17–1.89, *p* = 0.001, respectively, Table 2). In contrast, neither CEA nor CA 19–9 (measured before the start of adjuvant chemotherapy) correlated with OS in the group of resected PDAC patients. Optimal cut-off values of CA 19–9 (40.0 U/ml), CYFRA 21–1 (3.2 ng/ml), IL-6 (2.05 pg/ml), IL-10 (0.49 pg/ml), and TNF-α (2.95 pg/ml) could be determined by ROC curve and Youden Index analysis which allowed subdivision into two groups with a significant difference in OS as determined by Kaplan–Meier method and log rank test (for details see figure S5).

**Table 2:** Univariate analysis of the prognostic value of serum biomarker levels in patients with resected, locally advanced and metastatic PDAC

	Resected			Locally advanced			Metastatic		
	HR	95% CI	*p*	HR	95% CI	*p*	HR	95% CI	*p*
CA19-9	1.001	(0.999–1.004)	0.519	1.001	(1.0002–1.002)	0.017*	1.00001	(1.00001–1.00002)	0.0001**
CEA	1.16	(0.95–1.42)	0.140	0.99	(0.91–1.08)	0.828	1.002	(1.0004–1.004)	0.019*
CYFRA 21–1	1.45	(1.17–1.89)	0.001**	1.15	(0.93–1.42)	0.210	1.004	(1.001–1.006)	0.003**
HE4	1.00	(1.00–1.01)	0.614	1.00	(0.99–1.01)	0.900	1.0008	(1.00–1.002)	0.120
IFN-γ	0.98	(0.93–1.04)	0.589	1.08	(0.93–1.26)	0.339	1.004	(1.00–1.01)	0.307
IL-10	1.43	(1.09–1.87)	0.010*	7.74	(1.43–41.84)	0.017*	1.009	(0.82–1.4)	0.933
IL-6	1.12	(0.74–1.70)	0.585	1.30	(0.91–1.83)	0.149	1.001	(1.00–1.005)	0.663
IL-8	1.02	(1.00–1.04)	0.102	1.05	(0.99–1.11)	0.128	1.0006	(1.00–1.002)	0.476
TNF-α	1.31	(0.99–1.73)	0.058	1.44	(0.82–2.53)	0.205	1.006	(1.00–1.02)	0.180
PD-1	0.81	(0.51–1.30)	0.377	1.02	(0.76–1.37)	0.896	0.99	(0.94–1.04)	0.566
PD-L1	0.0003	(1.9 × 10^–10^ to 435)	0.261	9.6 × 10^–8^	(2 × 10^–20^ to 3 × 10^5^)	0.274	0.06	(0.0003–12.57)	0.300

Bold values indicate statistical significance

*HR* hazard ratio, *CI* confidence interval

*p < 0.05

** *p* < 0.01

In the group of locally advanced patients, higher IL-10 and higher CA 19–9 baseline levels correlated with poorer OS (HR 7.74, 95% CI 1.43–41.84, *p* = 0.017 and HR 1.001, 95% CI 1.0002–1.002, *p* = 0.017, respectively, Table 2). In this group, the cut-off values for CYFRA 21–1 (3.6 ng/ml), IL-8 (19.55 pg/ml), IL-10 (0.58 pg/ml), and IFN-γ (2.90 pg/ml) could determine two prognostic groups with a significant difference in OS (figure S6).

As expected, the most established PDAC biomarkers CEA, CA 19–9 and CYFRA 21–1 correlated with poorer prognosis in metastatic disease (HR 1.002, 95% CI 1.0004–1.004, *p* = 0.019, HR 1.00001, 95% CI 1.00001–1.00002, *p* = 0.0001 and HR 1.004, 95% CI 1.001–1.006, *p* = 0.003, respectively, Table 2). IL-10 did not seem to harbor any prognostic value in this patient group. We determined optimal cut-off values for CA 19–9 (1067 U/ml), CEA (8.4 ng/ml), CYFRA 21–1 (12.5 ng/ml), HE4 (82.2 pmol/l), IFNγ (3.3 pg/ml), IL-6 (3.3 pg/ml), IL-8 (29.65 pg/ml) and PD-L1 (0.008 ng/ml) levels in the metastatic group via ROC curve and Youden Index analysis. The cut-offs each allowed for division into two prognostic groups with significantly different OS (figure S7).

### Multivariate analysis of correlation of serological biomarkers with prognosis

When univariately significant parameters were included in multivariate Cox models for the endpoint OS, only CYFRA 21–1 remained an independent prognostic marker in the resected group (HR 1.37, 95% CI 1.06–1.77, *p* = 0.015) and only IL-10 in the locally advanced group (HR 10.01, 95% CI 1.58–63.19, *p* = 0.014). In the subgroup of patients with metastatic disease, CA 19–9 and CYFRA 21–1 harbored independent prognostic value (HR 1.00001, 95% CI 1.000003–100,002, *p* = 0.008 and HR 1.004, 95% CI 1.0008–1.006, *p* = 0.010, respectively) in the multivariate model.

## Discussion

In this analysis of biomarker serum levels from 162 PDAC patients treated at our comprehensive cancer center, we were able to investigate the prognostic role of various serological biomarkers across different stages of PDAC. In a first step, IL-1β, IL-2, IL-4, IL-12p70 and IL-13 were excluded from further analysis in this study, because more than 95% of measured values were below the lower level of quantification. These interleukins might have a role in PDAC biology nonetheless, however, pre-analytics, as well as the sensitivity of the diagnostic assay may have influenced the results. Previous reports have shown that IL-1β is produced by tumor cells and contributes to the immunosuppressive microenvironment in PDAC (Das et al. 2020). High IL-1β serum levels have been described to be associated with shorter OS (Mitsunaga et al. 2013; Piro et al. 2017). Furthermore, higher IL-4 serum levels have been found to be an independent prognostic factor for disease-free survival and significantly associated with shorter OS (Piro et al. 2017). Gabitass and co-workers demonstrated a significant increase in IL-13 levels in PDAC patients compared to healthy controls, and a positive correlation between IL-13 and myeloid-derived suppressor cell (MDSC) levels was found. Possibly due to their immunosuppressive potential, MDSC numbers were identified as an independent prognostic factor in PDAC patients (Gabitass et al. 2011). To further evaluate the prognostic role of the cytokines excluded in our study, protocol adaptation or use of an alternate, more sensitive assay may prove necessary.

HE4 is a protein better known as a relevant biomarker in ovarian cancer. We observed a significant correlation between age and HE4 serum levels which is in accordance with previous reports. HE4 levels have been described not only to increase with age in women and men (Hertlein et al. 2012; Moore et al. 2012; Cheng et al. 2020), but also correlate with renal function, inflammation, and hormonal levels (Qu et al. 2016). In our study, a cut-off value for HE4 serum levels that distinguished two prognostic groups could be determined for metastatic patients. While high expression levels of HE4 on PDAC cells have been associated with chemoresistance and poor prognosis (Ohkuma et al. 2021), to our knowledge, this has not yet been shown for HE4 serum levels. However, it must be noted that due to the correlation of HE4 serum levels with age, renal function and inflammation the prognostic value might be biased.

As expected, there were group differences in serum levels of most biomarkers between the resected and the metastatic patient subgroup. Interestingly, median CYFRA 21–1 and IL-8 serum levels significantly differed between the locally advanced and the metastatic subgroup, while not significantly differing between the resected and the locally advanced population. Both biomarkers each discriminated the locally advanced group from the metastatic group well as evaluated by area under the ROC curve. With CYFRA 21–1 and IL-8 serum levels apparently increased in metastatic disease, this information can complement imaging results in order to facilitate therapeutic decisions. IL-8 has been found to be produced by pancreatic cancer cells and correlate with metastatic potential and epithelial-mesenchymal transition (Chen et al. 2014; Matsuo et al. 2004), which may explain the significant difference in IL-8 serum levels between locally advanced and metastatic patients observed in this study.

The biomarkers evaluated in PDAC in clinical routine do not depend on the stage of the patient’s disease. In this study, univariate analysis revealed a significant correlation of (post-resection) IL-10 and CYFRA 21–1 with poorer outcome in patients with resected PDAC, a significant correlation of IL-10 and CA 19–9 with poorer outcome in locally advanced PDAC, as well as a significant correlation of CEA, CA 19–9, and CYFRA 21–1 with poorer outcome in metastatic disease. Therefore, the same biomarkers might have different prognostic values depending on the stage of the disease. When interpreting the results, it needs to be kept in mind that the HR is calculated for continuous variables, leading to HRs close to 1.0 but not including 1.0 when evaluating biomarkers with a broad variance. Interestingly, CA 19–9, the most established biomarker in PDAC, did not correlate with survival in the resected patient group, whereas IL-10 did. A small variance of IL-10 in this group resulted in a notably high HR of 10.01. Of note, the serum collection was performed before the start of adjuvant chemotherapy in our patient cohort and not before surgery; thus, the pancreatic tumor had already been removed when blood for the serum analyses was drawn. However, a similar observation could be demonstrated in the locally advanced group in this study, for which the independent prognostic role of IL-10 could be confirmed in the multivariate analysis. Higher levels of IL-10 have been described to be associated with poor survival before (Feng et al. 2018). This observation could possibly be explained by the known immunosuppressive effect of IL-10 in cancer (Sideras et al. 2014). The significant association of CYFRA 21–1 with OS in the resected and the metastatic group was maintained in multivariate analysis. Our group has previously described CYFRA 21–1 to significantly correlate with OS in advanced pancreatic cancer (Boeck et al. 2013).

In the current study, we did not only evaluate the prognostic value of different biomarkers, but we also determined cut-off values of the serum levels, which allowed the division of the patients into two prognostic groups. Cut-off values can be easily applied in clinical routine and support decision-making based on estimated prognosis. For most biomarkers, serum levels above the cut-off value marked belonging to the group with a shorter OS. However, for IFN-γ and PD-L1 in the metastatic setting, the group with a better prognosis had serum levels above the cut-off value. IFN-γ has been described to inhibit the proliferation and migration of PDAC (Lange et al. 2011; Zhang et al. 2018), however, high PD-L1 expression on pancreatic cancer cells has been associated with a poor prognosis in multiple previous studies (Nomi et al. 2007; Gao et al. 2018; Hu et al. 2019; Zhao and Cao 2020). But the same might not hold true for serum levels of PD-L1. We have previously reported that PD-L1 serum levels do not correlate with tumoral PD-L1 expression, and we did not find high serum PD-L1 levels to be an adverse prognostic marker (Kruger et al. 2017), which again was verified in this study.

With this study being monocentric and retrospective, a prospective validation of the data is recommended. The small number of patients in the locally advanced group is a limitation of this study and especially the findings related to this group call for further validation in a larger patient cohort. When interpreting the presented data, it should be noted that in contrast to the locally advanced and metastatic PDAC group, the resected patient group has already undergone resection of the tumor at the time point of the blood draw. This way, the tumoral burden is significantly lower, while there might still be some postoperative cytokine changes obscuring the measurements. Furthermore, a potential selection bias could have occurred because only patients treated at our comprehensive cancer center who also consented to the study were included. However, with patient outcomes matching what is to be expected from the literature, the patient population seems to be representative in this regard. Due to limited amounts of serum samples, we chose one commercially available panel diagnostic kit that included a variety of inflammatory biomarkers of interest, however, this way, other potentially relevant biomarkers had to be omitted and may be included in further studies. Despite the mentioned limitations, this study provides an important understanding of the role of an extensive biomarker panel in PDAC.

In conclusion, this study demonstrated that depending on the stage of pancreatic cancer, other biomarkers than CA 19–9 might provide helpful prognostic information and support patient stratification. CYFRA 21–1 and IL-8 have been identified to discriminate metastatic patients well from locally advanced patients, potentially adding information to imaging results and facilitating decisions in clinical routine. Furthermore, useful cut-off values have been calculated for various biomarker serum levels to easily determine prognostic groups with significantly different OS.

## Supplementary Information

Below is the link to the electronic supplementary material.

Fig. S1 Study design Supplementary file1 (TIF 1343 KB)

Figure S2 Therapy after study inclusion Supplementary file2 (TIF 1225 KB)

Fig. S3 Distribution of measured serum biomarker levels logarithmized to base 10 and normalized to the median Supplementary file3 (TIF 1343 KB)

Fig. S4 a) Correlation of HE4 serum levels and age, both logarithmized to base 10 Supplementary file4 (TIF 767 KB)

b) Correlation of TNF-α serum levels and age, both logarithmized to base 10 Supplementary file5 (TIF 739 KB)

Fig. S5 Post tumor resection with curative intention: Receiver operator characteristics (ROC) curves and Youden index for determination of a serum biomarker level threshold differentiating patients with a longer or shorter survival time compared to the subgroup-specific median. The ROC curves show the true-positive rates versus the false-positive rates assuming different threshold values. Thresholds with maximal sensitivity and specificity determined by the maximal Youden index are marked by a red dot. Kaplan-Meier curves and the associated p-value determined by log-rank test for any difference in the survival rates are given. Supplementary file6 (TIF 5569 KB)

Fig. S6 Locally advanced pancreatic cancer: Receiver operator characteristics (ROC) curves and Youden index for determination of a serum biomarker level threshold differentiating patients with a longer or shorter survival time compared to the subgroup-specific median. The ROC curves show the true-positive rates versus the false-positive rates assuming different threshold values. Thresholds with maximal sensitivity and specificity determined by the maximal Youden index are marked by a red dot. Kaplan-Meier curves and the associated p-value determined by log-rank test for any difference in the survival rates are given. Supplementary file7 (TIF 4491 KB)

Fig. S7 Metastatic pancreatic cancer: Receiver operator characteristics (ROC) curves and Youden index for determination of a serum biomarker level threshold differentiating patients with a longer or shorter survival time compared to the subgroup-specific median. The ROC curves show the true-positive rates versus the false-positive rates assuming different threshold values. Thresholds with maximal sensitivity and specificity determined by the maximal Youden index are marked by a red dot. Kaplan-Meier curves and the associated p-value determined by log-rank test for any difference in the survival rates are given. Supplementary file8 (TIF 4739 KB)

Supplementary file9 (TIF 4684 KB)

Supplementary file10 (DOCX 14 KB)

## Data Availability

The datasets generated and analysed during the current study are available from the corresponding author on reasonable request.

## References

[CR1] Boeck S, Stieber P, Holdenrieder S, Wilkowski R, Heinemann V (2006). Prognostic and therapeutic significance of carbohydrate antigen 19–9 as tumor marker in patients with pancreatic cancer. Oncology.

[CR2] Boeck S, Haas M, Laubender RP, Kullmann F, Klose C, Bruns CJ, Wilkowski R, Stieber P, Holdenrieder S, Buchner H, Mansmann U, Heinemann V (2010). Application of a time-varying covariate model to the analysis of CA 19–9 as serum biomarker in patients with advanced pancreatic cancer. Clin Cancer Res.

[CR3] Boeck S, Wittwer C, Heinemann V, Haas M, Kern C, Stieber P, Nagel D, Holdenrieder S (2013). Cytokeratin 19-fragments (CYFRA 21–1) as a novel serum biomarker for response and survival in patients with advanced pancreatic cancer. Br J Cancer.

[CR4] Chen L, Fan J, Chen H, Meng Z, Chen Z, Wang P, Liu L (2014). The IL-8/CXCR1 axis is associated with cancer stem cell-like properties and correlates with clinical prognosis in human pancreatic cancer cases. Sci Rep.

[CR5] Cheng HY, Zeng L, Ye X, Ma RQ, Tang ZJ, Chu HL, Zhao YM, Zhu LR, Gao YN, Chang XH, Cui H (2020). Age and menopausal status are important factors influencing the serum human epididymis secretory protein 4 level: a prospective cross-sectional study in healthy Chinese people. Chin Med J (Engl).

[CR6] Chiorean EG, Von Hoff DD, Reni M, Arena FP, Infante JR, Bathini VG, Wood TE, Mainwaring PN, Muldoon RT, Clingan PR, Kunzmann V, Ramanathan RK, Tabernero J, Goldstein D, McGovern D, Lu B, Ko A (2016). CA19-9 decrease at 8 weeks as a predictor of overall survival in a randomized phase III trial (MPACT) of weekly nab-paclitaxel plus gemcitabine versus gemcitabine alone in patients with metastatic pancreatic cancer. Ann Oncol.

[CR7] Conroy T, Desseigne F, Ychou M, Bouché O, Guimbaud R, Bécouarn Y, Adenis A, Raoul JL, Gourgou-Bourgade S, de la Fouchardière C, Bennouna J, Bachet JB, Khemissa-Akouz F, Péré-Vergé D, Delbaldo C, Assenat E, Chauffert B, Michel P, Montoto-Grillot C, Ducreux M (2011). FOLFIRINOX versus gemcitabine for metastatic pancreatic cancer. N Engl J Med.

[CR8] Das S, Shapiro B, Vucic EA, Vogt S, Bar-Sagi D (2020). Tumor cell-derived IL1β promotes desmoplasia and immune suppression in pancreatic cancer. Cancer Res.

[CR9] Feng L, Qi Q, Wang P, Chen H, Chen Z, Meng Z, Liu L (2018). Serum levels of IL-6, IL-8, and IL-10 are indicators of prognosis in pancreatic cancer. J Int Med Res.

[CR10] Gabitass RF, Annels NE, Stocken DD, Pandha HA, Middleton GW (2011). Elevated myeloid-derived suppressor cells in pancreatic, esophageal and gastric cancer are an independent prognostic factor and are associated with significant elevation of the Th2 cytokine interleukin-13. Cancer Immunol Immunother.

[CR11] Gao HL, Liu L, Qi ZH, Xu HX, Wang WQ, Wu CT, Zhang SR, Xu JZ, Ni QX, Yu XJ (2018). The clinicopathological and prognostic significance of PD-L1 expression in pancreatic cancer: a meta-analysis. Hepatobiliary Pancreat Dis Int.

[CR12] Haas M, Heinemann V, Kullmann F, Laubender RP, Klose C, Bruns CJ, Holdenrieder S, Modest DP, Schulz C, Boeck S (2013). Prognostic value of CA 19–9, CEA, CRP, LDH and bilirubin levels in locally advanced and metastatic pancreatic cancer: results from a multicenter, pooled analysis of patients receiving palliative chemotherapy. J Cancer Res Clin Oncol.

[CR13] Hertlein L, Stieber P, Kirschenhofer A, Krocker K, Nagel D, Lenhard M, Burges A (2012). Human epididymis protein 4 (HE4) in benign and malignant diseases. Clin Chem Lab Med.

[CR14] Hu Y, Chen W, Yan Z, Ma J, Zhu F, Huo J (2019). Prognostic value of PD-L1 expression in patients with pancreatic cancer: a PRISMA-compliant meta-analysis. Medicine (baltimore).

[CR15] Kruger S, Legenstein ML, Rösgen V, Haas M, Modest DP, Westphalen CB, Ormanns S, Kirchner T, Heinemann V, Holdenrieder S, Boeck S (2017). Serum levels of soluble programmed death protein 1 (sPD-1) and soluble programmed death ligand 1 (sPD-L1) in advanced pancreatic cancer. Oncoimmunology.

[CR16] Lange F, Rateitschak K, Fitzner B, Pöhland R, Wolkenhauer O, Jaster R (2011). Studies on mechanisms of interferon-gamma action in pancreatic cancer using a data-driven and model-based approach. Mol Cancer.

[CR17] Liu L, Xu H, Wang W, Wu C, Chen Y, Yang J, Cen P, Xu J, Liu C, Long J, Guha S, Fu D, Ni Q, Jatoi A, Chari S, McCleary-Wheeler AL, Fernandez-Zapico ME, Li M, Yu X (2015). A preoperative serum signature of CEA+/CA125+/CA19-9 ≥ 1000 U/mL indicates poor outcome to pancreatectomy for pancreatic cancer. Int J Cancer.

[CR18] Matsuo Y, Sawai H, Funahashi H, Takahashi H, Sakamoto M, Yamamoto M, Okada Y, Hayakawa T, Manabe T (2004). Enhanced angiogenesis due to inflammatory cytokines from pancreatic cancer cell lines and relation to metastatic potential. Pancreas.

[CR19] Mitsunaga S, Ikeda M, Shimizu S, Ohno I, Furuse J, Inagaki M, Higashi S, Kato H, Terao K, Ochiai A (2013). Serum levels of IL-6 and IL-1β can predict the efficacy of gemcitabine in patients with advanced pancreatic cancer. Br J Cancer.

[CR20] Moore RG, Miller MC, Eklund EE, Lu KH, Bast RC, Lambert-Messerlian G (2012). Serum levels of the ovarian cancer biomarker HE4 are decreased in pregnancy and increase with age. Am J Obstet Gynecol.

[CR21] Nomi T, Sho M, Akahori T, Hamada K, Kubo A, Kanehiro H, Nakamura S, Enomoto K, Yagita H, Azuma M, Nakajima Y (2007). Clinical significance and therapeutic potential of the programmed death-1 ligand/programmed death-1 pathway in human pancreatic cancer. Clin Cancer Res.

[CR22] Ohkuma R, Yada E, Ishikawa S, Komura D, Kubota Y, Hamada K, Horiike A, Ishiguro T, Hirasawa Y, Ariizumi H, Shida M, Watanabe M, Onoue R, Ando K, Tsurutani J, Yoshimura K, Sasada T, Aoki T, Murakami M, Norose T, Ohike N, Takimoto M, Kobayashi S, Tsunoda T, Wada S (2021). High levels of human epididymis protein 4 mRNA and protein expression are associated with chemoresistance and a poor prognosis in pancreatic cancer. Int J Oncol.

[CR23] Padoan A, Plebani M, Basso D (2019). Inflammation and pancreatic cancer: focus on metabolism, cytokines, and immunity. Int J Mol Sci.

[CR24] Piro G, Simionato F, Carbone C, Frizziero M, Malleo G, Zanini S, Casolino R, Santoro R, Mina MM, Zecchetto C, Merz V, Scarpa A, Bassi C, Tortora G, Melisi D (2017). A circulating T(H)2 cytokines profile predicts survival in patients with resectable pancreatic adenocarcinoma. Oncoimmunology.

[CR25] Qu W, Li J, Duan P, Tang Z, Guo F, Chen H, Zhu X, Jiang SW (2016). Physiopathological factors affecting the diagnostic value of serum HE4-test for gynecologic malignancies. Expert Rev Mol Diagn.

[CR26] Rahib L, Smith BD, Aizenberg R, Rosenzweig AB, Fleshman JM, Matrisian LM (2014). Projecting cancer incidence and deaths to 2030: the unexpected burden of thyroid, liver, and pancreas cancers in the United States. Cancer Res.

[CR27] Sideras K, Braat H, Kwekkeboom J, van Eijck CH, Peppelenbosch MP, Sleijfer S, Bruno M (2014). Role of the immune system in pancreatic cancer progression and immune modulating treatment strategies. Cancer Treat Rev.

[CR28] Siegel RL, Miller KD, FuchsJemal HEA (2021). Cancer statistics, 2021. CA Cancer J Clin.

[CR29] Song J, Sokoll LJ, Pasay JJ, Rubin AL, Li H, Bach DM, Chan DW, Zhang Z (2019). Identification of serum biomarker panels for the early detection of pancreatic cancer. Cancer Epidemiol Biomarkers Prev.

[CR30] van Manen L, Groen JV, Putter H, Vahrmeijer AL, Swijnenburg RJ, Bonsing BA, Mieog JSD (2020). Elevated CEA and CA19-9 serum levels independently predict advanced pancreatic cancer at diagnosis. Biomarkers.

[CR31] Von Hoff DD, Ervin T, Arena FP, Chiorean EG, Infante J, Moore M, Seay T, Tjulandin SA, Ma WW, Saleh MN, Harris M, Reni M, Dowden S, Laheru D, Bahary N, Ramanathan RK, Tabernero J, Hidalgo M, Goldstein D, Van Cutsem E, Wei X, Iglesias J, Renschler MF (2013). Increased survival in pancreatic cancer with nab-paclitaxel plus gemcitabine. N Engl J Med.

[CR32] Zhang M, Ding G, Zhou L, Shen T, Xu X, Zhao T, Jia S, Cao L (2018). Interferon gamma inhibits CXCL8-induced proliferation and migration of pancreatic cancer BxPC-3 cell line via a RhoGDI2/Rac1/NF-κB signalling pathway. J Interferon Cytokine Res.

[CR33] Zhao L, Cao Y (2020). PD-L1 expression level displays a positive correlation with immune response in pancreatic cancer. Dis Markers.

