# Supplementary figures and images for: Serum biomarker panel diagnostics in pancreatic ductal adenocarcinoma: the clinical utility of soluble interleukins, IFN-γ, TNF-α and PD-1/PD-L1 in comparison to established serum tumor markers
Source: J Cancer Res Clin Oncol. 2022 Jun 23;149(6):2463–74. doi: 10.1007/s00432-022-04112-z (PMC10130000; doi:10.1007/s00432-022-04112-z)

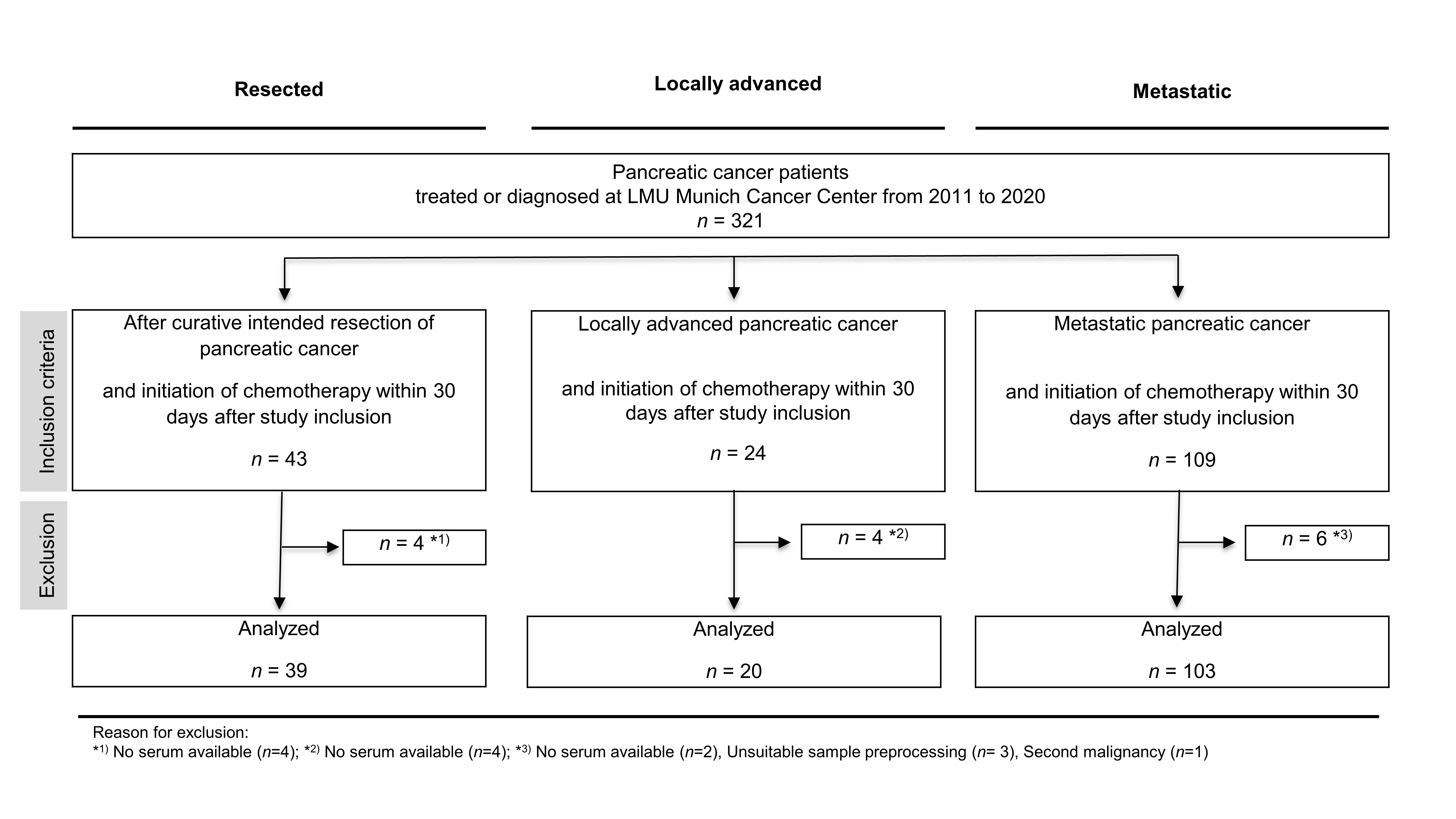

Supplement: Supplementary file 1 — Fig. S1 Study design Supplementary file1 (TIF 1343 KB) [file 432_2022_4112_MOESM1_ESM.tif]

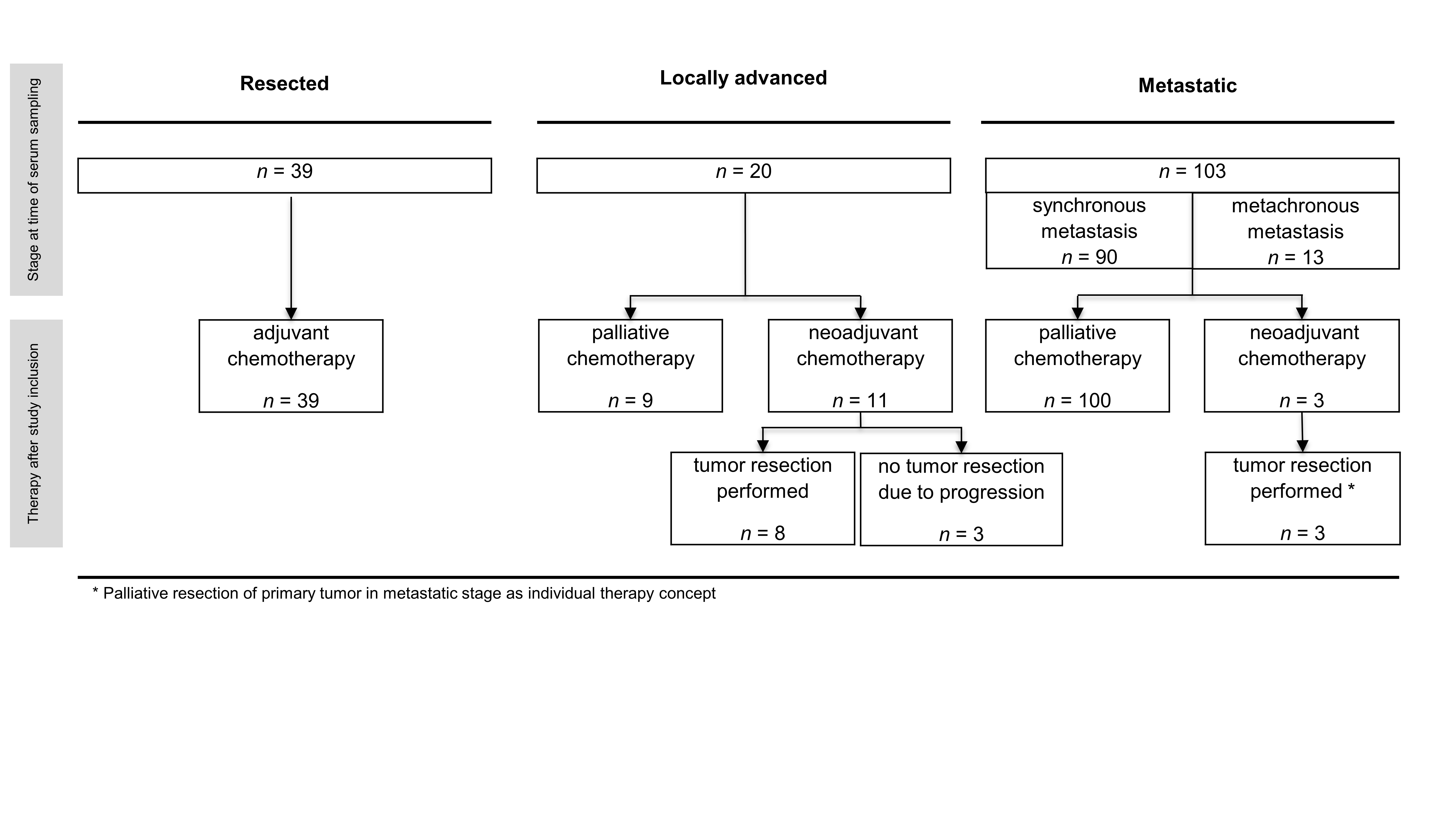

Supplement: Supplementary file 2 — Figure S2 Therapy after study inclusion Supplementary file2 (TIF 1225 KB) [file 432_2022_4112_MOESM2_ESM.tif]

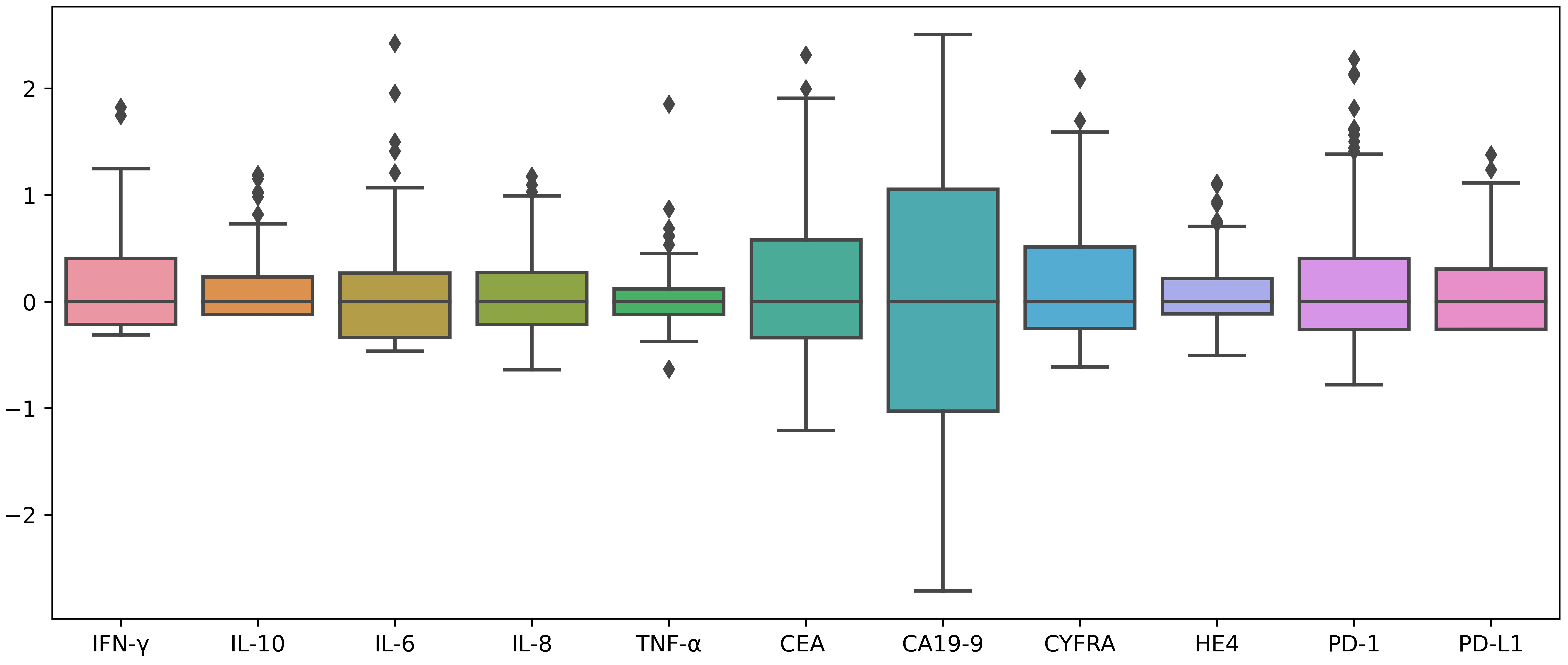

Supplement: Supplementary file 3 — Fig. S3 Distribution of measured serum biomarker levels logarithmized to base 10 and normalized to the median Supplementary file3 (TIF 1343 KB) [file 432_2022_4112_MOESM3_ESM.tif]

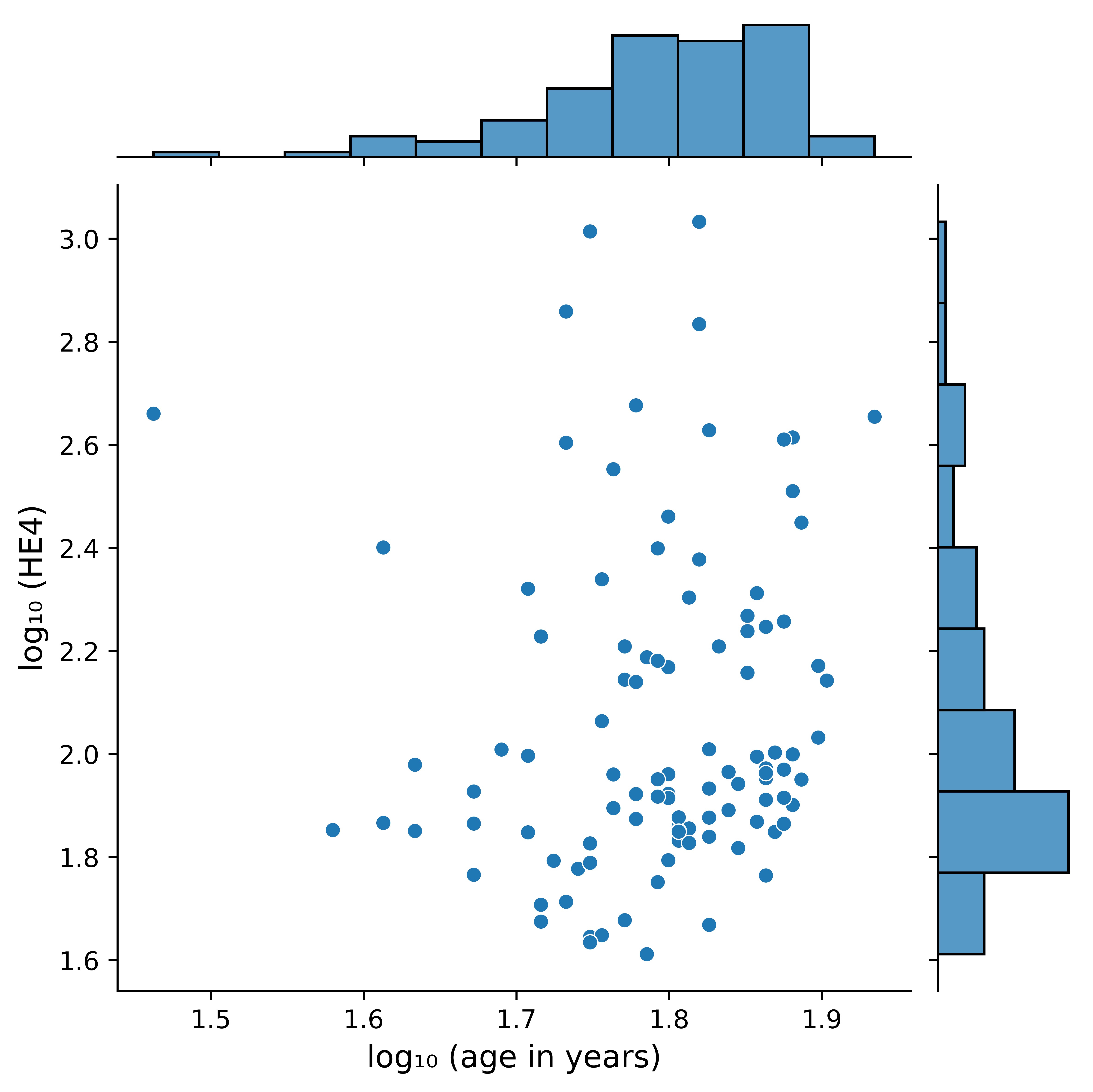

Supplement: Supplementary file 4 — Fig. S4 a) Correlation of HE4 serum levels and age, both logarithmized to base 10 Supplementary file4 (TIF 767 KB) [file 432_2022_4112_MOESM4_ESM.tif]

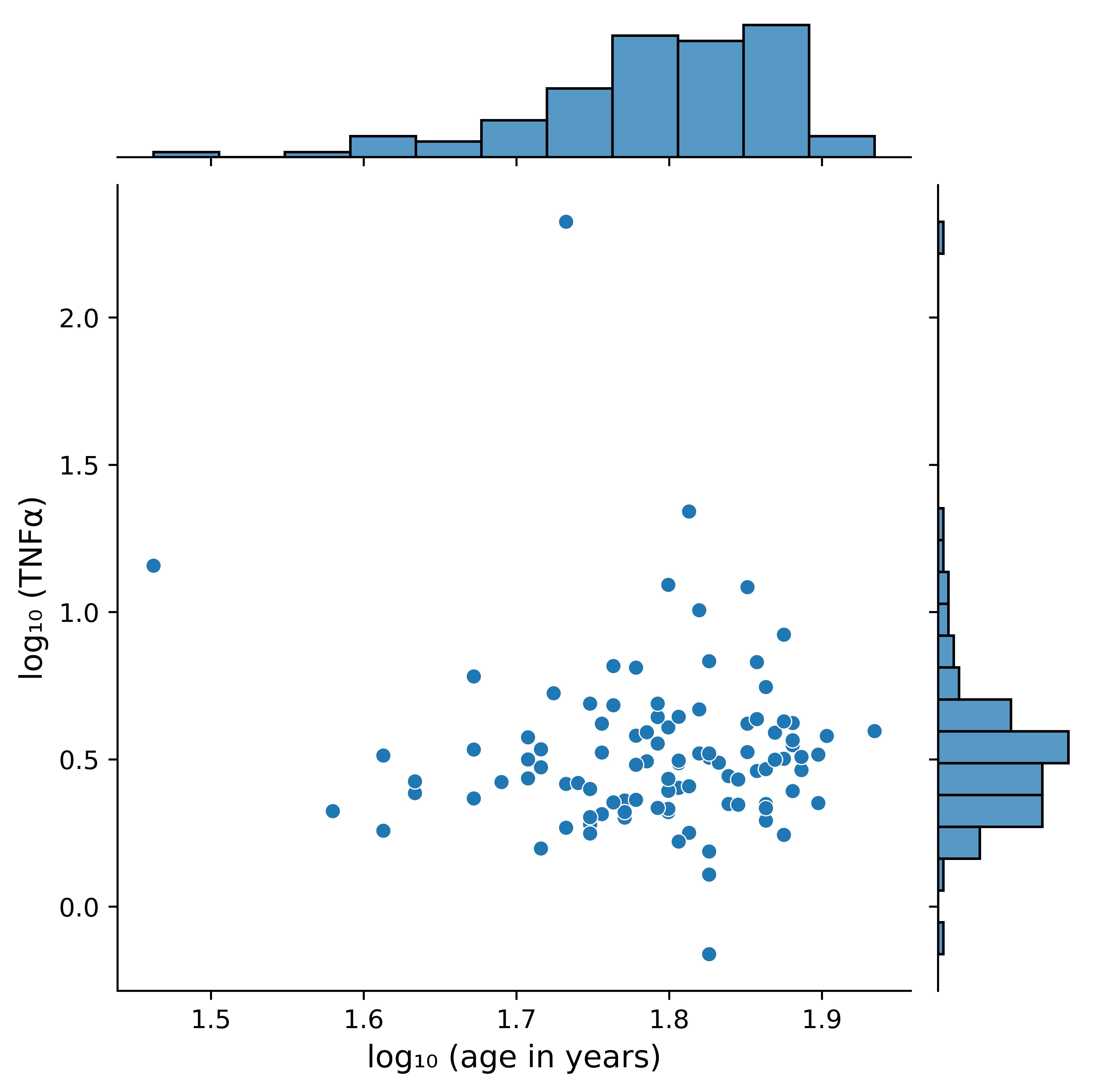

Supplement: Supplementary file 5 — b) Correlation of TNF-α serum levels and age, both logarithmized to base 10 Supplementary file5 (TIF 739 KB) [file 432_2022_4112_MOESM5_ESM.tif]

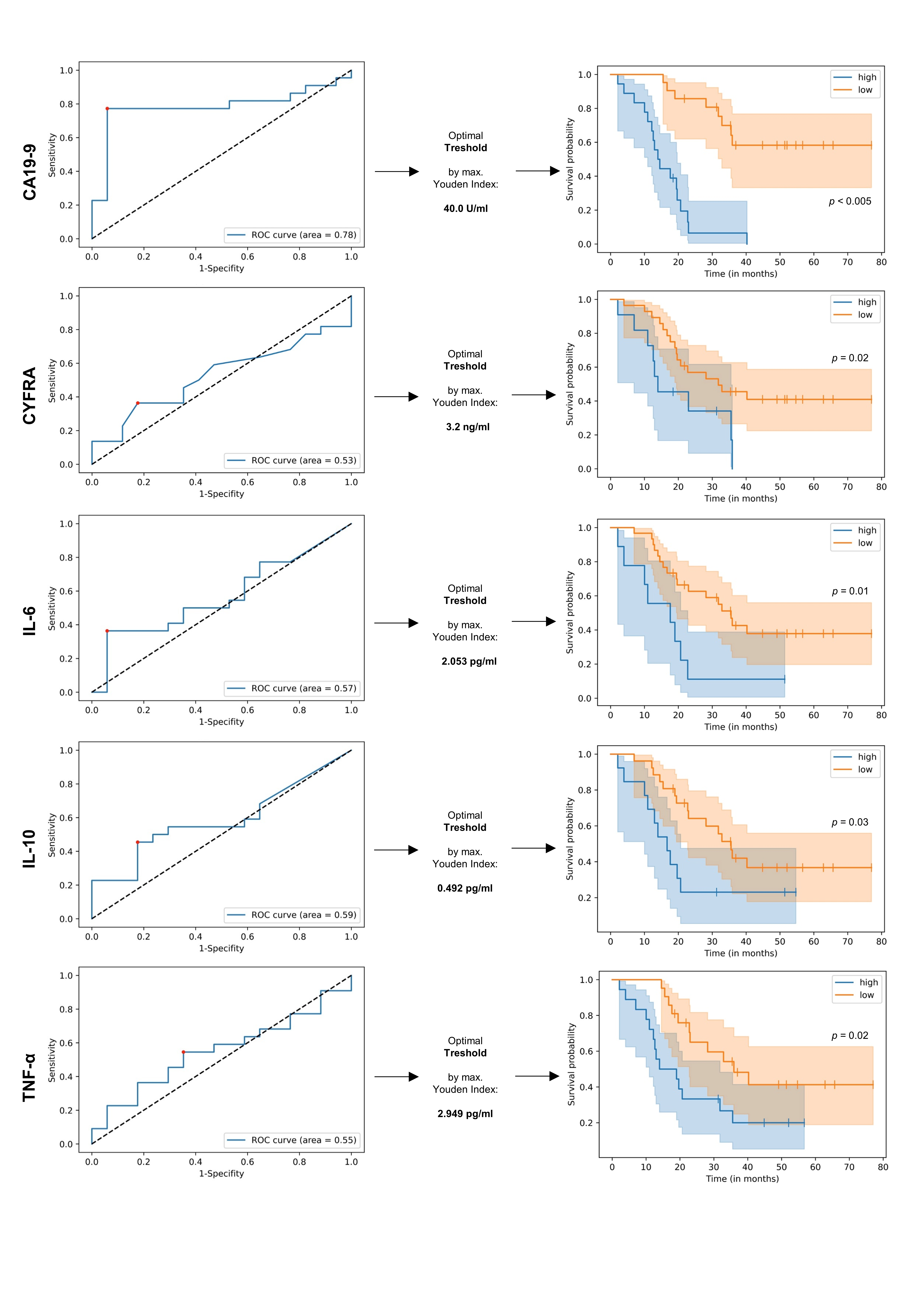

Supplement: Supplementary file 6 — Fig. S5 Post tumor resection with curative intention: Receiver operator characteristics (ROC) curves and Youden index for determination of a serum biomarker level threshold differentiating patients with a longer or shorter survival time compared to the subgroup-specific median. The ROC curves show the true-positive rates versus the false-positive rates assuming different threshold values. Thresholds with maximal sensitivity and specificity determined by the maximal Youden index are marked by a red dot. Kaplan-Meier curves and the associated p-value determined by log-rank test for any difference in the survival rates are given. Supplementary file6 (TIF 5569 KB) [file 432_2022_4112_MOESM6_ESM.tif]

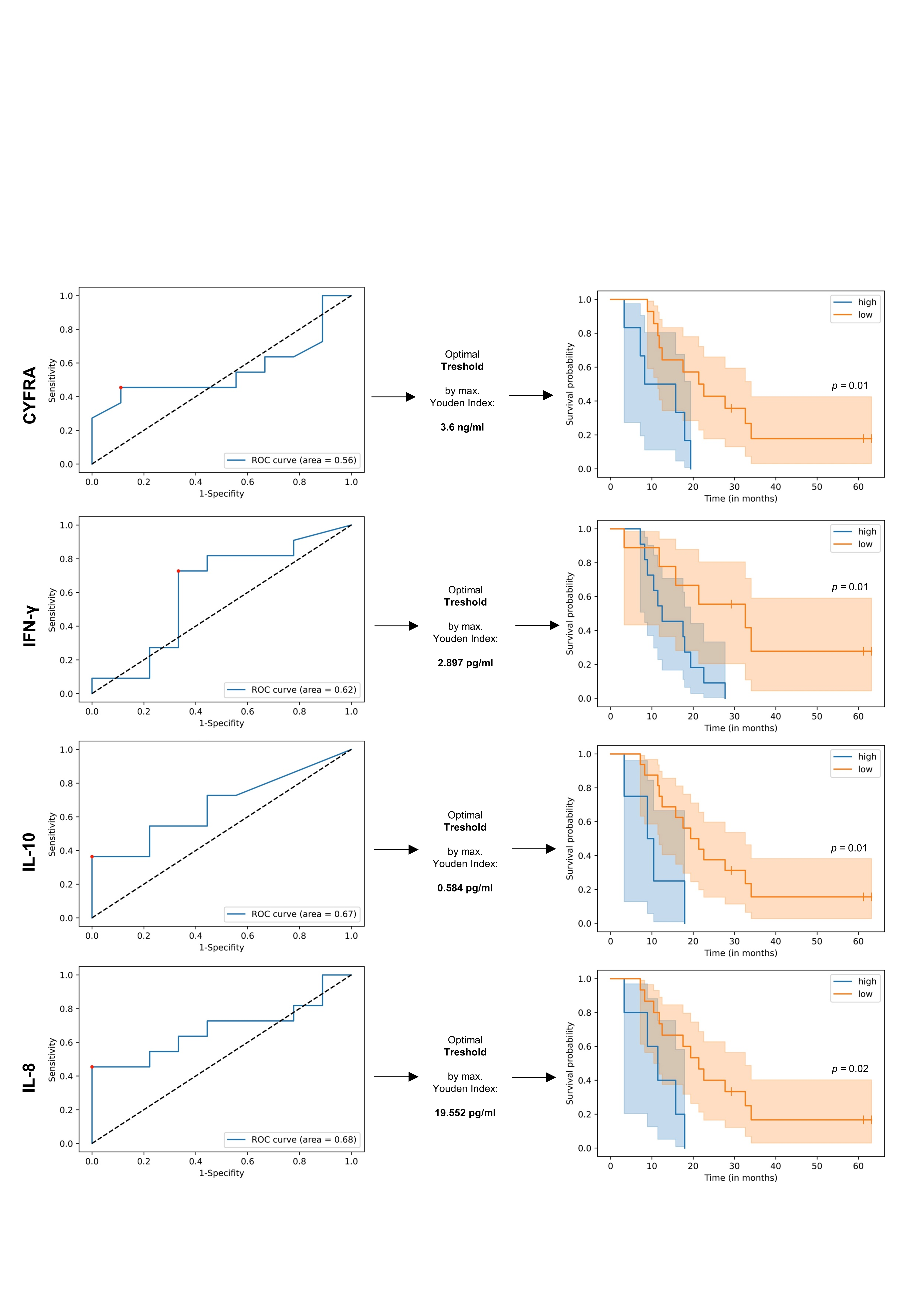

Supplement: Supplementary file 7 — Fig. S6 Locally advanced pancreatic cancer: Receiver operator characteristics (ROC) curves and Youden index for determination of a serum biomarker level threshold differentiating patients with a longer or shorter survival time compared to the subgroup-specific median. The ROC curves show the true-positive rates versus the false-positive rates assuming different threshold values. Thresholds with maximal sensitivity and specificity determined by the maximal Youden index are marked by a red dot. Kaplan-Meier curves and the associated p-value determined by log-rank test for any difference in the survival rates are given. Supplementary file7 (TIF 4491 KB) [file 432_2022_4112_MOESM7_ESM.tif]

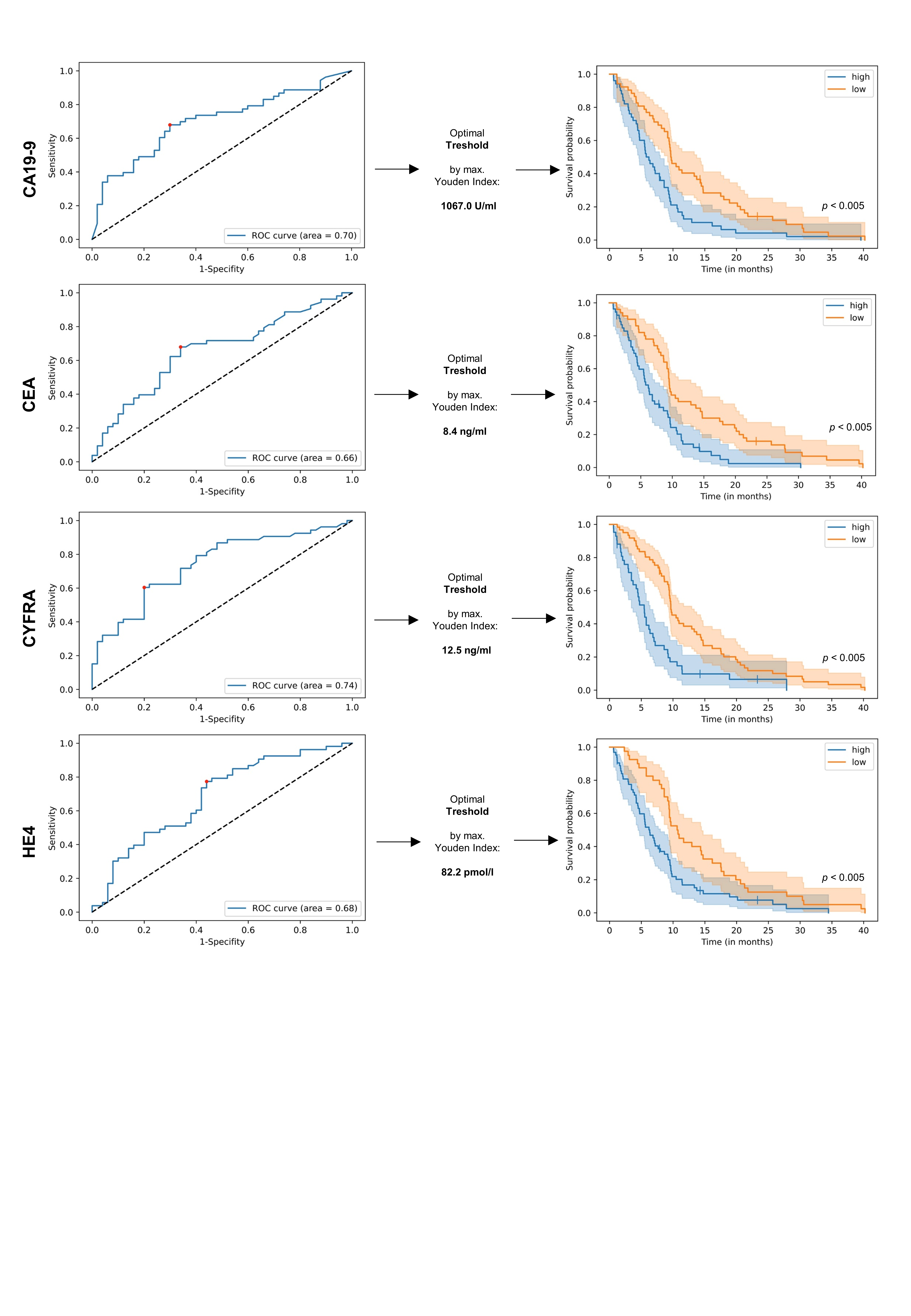

Supplement: Supplementary file 8 — Fig. S7 Metastatic pancreatic cancer: Receiver operator characteristics (ROC) curves and Youden index for determination of a serum biomarker level threshold differentiating patients with a longer or shorter survival time compared to the subgroup-specific median. The ROC curves show the true-positive rates versus the false-positive rates assuming different threshold values. Thresholds with maximal sensitivity and specificity determined by the maximal Youden index are marked by a red dot. Kaplan-Meier curves and the associated p-value determined by log-rank test for any difference in the survival rates are given. Supplementary file8 (TIF 4739 KB) [file 432_2022_4112_MOESM8_ESM.tif]

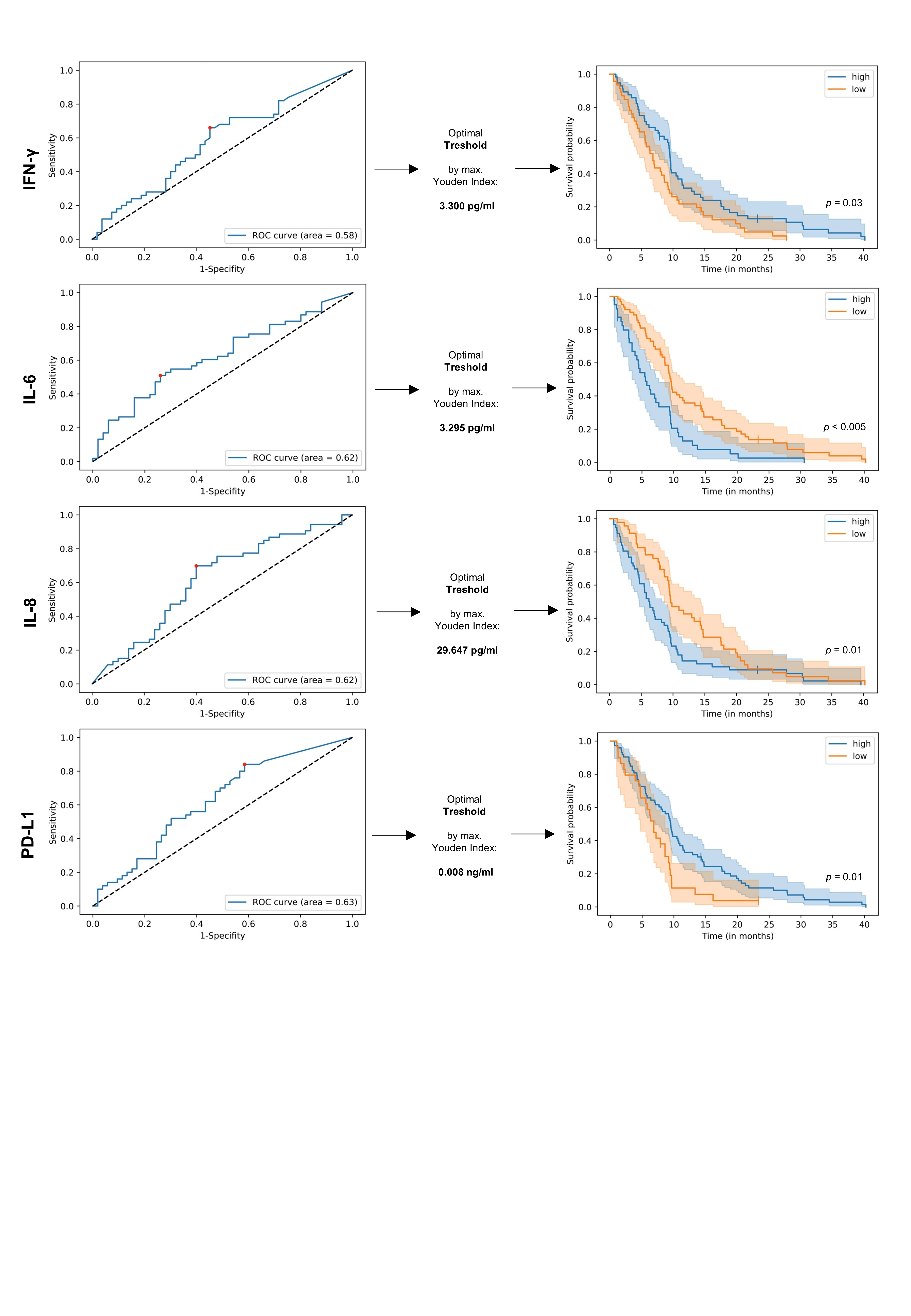

Supplement: Supplementary file 9 — Supplementary file9 (TIF 4684 KB) [file 432_2022_4112_MOESM9_ESM.tif]
