# Supplementary material for: Serum biomarker panel diagnostics in pancreatic ductal adenocarcinoma: the clinical utility of soluble interleukins, IFN-γ, TNF-α and PD-1/PD-L1 in comparison to established serum tumor markers
Source: J Cancer Res Clin Oncol. 2022 Jun 23;149(6):2463–74. doi: 10.1007/s00432-022-04112-z (PMC10130000; doi:10.1007/s00432-022-04112-z)
Supplement: Supplementary file 10 — Supplementary file10 (DOCX 14 KB) [file 432_2022_4112_MOESM10_ESM.docx]

**Table S1** Lower and upper limits of quantification

| **Biomarker** | **LLOQ** | **ULOQ** | **Values below LLOQ** | **Values above ULOQ** | **Values within LOQ** |
| --- | --- | --- | --- | --- | --- |
| **IL-1β** | 0.646 pg/ml | 375 pg/ml | 157 (97%) | 0 (0%) | 5 ( 3%) |
| **IL-2** | 0.89 pg/ml | 938 pg/ml | 155 (96%) | 0 (0%) | 7 ( 4%) |
| **IL-4** | 0.218 pg/ml | 158 pg/ml | 159 (98%) | 0 (0%) | 3 ( 2%) |
| **IL-6** | 0.633 pg/ml | 488 pg/ml | 20 (12%) | 1 (1%) | 141 (87%) |
| **IL-8** | 0.591 pg/ml | 375 pg/ml | 0 ( 0%) | 9 (6%) | 153 (94%) |
| **IL-10** | 0.298 pg/ml | 233 pg/ml | 52 (32%) | 0 (0%) | 110 (68%) |
| **IL-12p70** | 1.22 pg/ml | 315 pg/ml | 158 (98%) | 0 (0%) | 4 ( 2%) |
| **IL-13** | 4.21 pg/ml | 353 pg/ml | 159 (98%) | 0 (0%) | 3 ( 2%) |
| **IFN-γ** | 1.76 pg/ml | 938 pg/ml | 29 (18%) | 0 (0%) | 133 (82%) |
| **TNF-α** | 0.69 pg/ml | 248 pg/ml | 3 ( 2%) | 0 (0%) | 159 (98%) |
| **CA19-9** | 0.6 U/ml | 100000 U/ml | 8 ( 5%) | 6 (4%) | 148 (91%) |
| **CEA** | 0.2 ng/ml | 1000 ng/ml | 0 ( 0%) | 1 (1%) | 161 (99%) |
| **CYFRA 21-1** | 0.1 ng/ml | 500 ng/ml | 0 ( 0%) | 1 (1%) | 161 (99%) |
| **HE4** | 15 pmol/l | 1500 pmol/l | 0 ( 0%) | 0 (0%) | 162 (100%) |
| **PD-1** | 0.0073 ng/ml | 30 ng/ml | 0 ( 0%) | 1 (1%) | 161 (99%) |
| **PD-L1** | 0.0073 ng/ml | 30 ng/ml | 47 (29%) | 0 (0%) | 115 (71%) |

*LLOQ* lower limit of quantification; *ULOQ* upper limit of quantification; *LOQ* limits of quantification
